# Can Adoptive Immunotherapy With Hepatitis E Virus (HEV)–Specific T Cells Address the Unmet Need in Refractory Chronic HEV Infection?

**DOI:** 10.1093/ofid/ofaf231

**Published:** 2025-05-27

**Authors:** Temi Lampejo

**Affiliations:** Faculty of Medicine and Life Sciences, King's College London, London, UK; Department of Infection Sciences, King's College Hospital, London, UK

**Keywords:** hepatitis E virus, HEV, persistent, refractory, virus-specific T cells

## Abstract

Chronic hepatitis E virus (HEV) infection, which primarily affects the immunocompromised, can rapidly progress to liver fibrosis and cirrhosis if untreated. However, current therapeutic options are extremely limited and have significant adverse effects. Over the past decade, virus-specific T-cell therapy has shown promise as an alternative safe and effective treatment strategy for other refractory viral infections such as cytomegalovirus, adenovirus, and polyomavirus infections in hematopoietic stem cell and solid organ transplant recipients. Given the key role of T lymphocytes in the control of HEV replication and the fact that HEV-specific T-cell responses are typically diminished in immunosuppressed patients with persistent HEV infection, adoptive immunotherapy with HEV-specific T cells could serve as a novel addition to the HEV treatment repertoire, which is in dire need of expansion.

## WHICH PATIENT POPULATIONS ARE AT RISK OF DEVELOPING CHRONIC HEV INFECTION?

Hepatitis E virus (HEV) is a small, nonenveloped (in faeces and bile but with a cell-derived lipid quasi-envelope when circulating in blood) RNA virus belonging the *Hepevirus* genus in the *Hepeviridae* family [1]. There are at least 4 HEV genotypes; genotypes 1 and 2 (both typically acquired through contaminated water) are restricted to humans, whereas genotypes 3 and 4 are known to circulate among various animals including pigs, wild boars, and deer, occasionally being transmitted to humans, that is, zoonotic transmission (eg, through eating undercooked pork/meat). Globally, there are an estimated 20 million human HEV infections each year, leading to an estimated 3.3 million cases of symptomatic HEV infection [2]. Following an incubation period of 2 to 10 weeks (5 to 6 weeks on average), immunocompetent individuals acutely infected with HEV typically eradicate their infection via host immune clearance, whereas the development of chronic HEV infection, almost exclusively due to HEV genotype 3, is typically limited to those with immunodeficiency.

The human host immune response to HEV is considered a combination of innate, adaptive humoral, and cellular responses [3]. Although HEV-specific antibodies develop in response to natural infection, they have not been clearly shown to have a major role in viral clearance of acute infection [4]. Additionally, they do not provide sterilizing immunity, and their main target, the ORF 2 capsid protein, due to its quasi-enveloped configuration, is able to prevent recognition by neutralizing HEV-specific antibodies [3]. It is evident that many patients with persistent HEV infection can produce HEV-specific antibodies without eliminating the virus [5]. Moreover, convalescent plasma containing high levels of anti-HEV immunoglobulin G (derived from donors who had a recent acute HEV infection) was unsuccessful in the treatment of a patient with persistent refractory HEV infection despite multiple infusions, and no significant variations in the patient's plasma HEV RNA levels were observed following the infusions [6]. T-cell responses are now considered to play the dominant role in achieving control of HEV infection and viral clearance and thus are a major determinant as to whether progression to chronic infection occurs [3, 4]. Following the early, acute phase of HEV infection, HEV is thought to be largely controlled by the interaction of CD4+ helper and CD8+ cytotoxic T cells [3]. T-cell exhaustion and diminished CD4+ and CD8+ T-cell responses are seen in the majority immunosuppressed individuals with chronic HEV infection [7, 8].

Chronic HEV infections are seen in solid organ transplant (SOT) recipients, individuals with advanced HIV infection, and patients treated for hematological malignancies including hematopoietic stem cell transplant (HSCT) recipients and, more recently, chimeric antigen receptor (CAR) T-cell therapy recipients [9, 10]. Additionally, HEV reinfections have been reported in SOT recipients who were HEV seropositive before transplantation, although it is unknown whether reinfection was with a different HEV genotype or if there may have been an unidentified HEV reservoir, that is, reactivation rather than reinfection [11]. The latter has been reported in the post-HSCT setting, with HEV reactivation confirmed by sequence analysis [12]. A meta-analysis reported an estimated pooled prevalence of de novo HEV infection of 5.1% in SOT recipients [13]. Large cohort studies have shown evolution of HEV to chronic infection in ∼66% of solid organ transplant recipients and 34% of patients with hematological malignancies (46% of allogeneic HSCT recipients) [14, 15]. Chronic HEV infection can progress to fibrosis and even cirrhosis at varying rates, and an alarming 10% of SOT recipients progress to cirrhosis within 2 years if not successfully treated [16]. In addition, rare extrahepatic complications can develop including neurological, renal, and hematological disorders [17].

## WHAT ARE THE CURRENT THERAPEUTIC STRATEGIES FOR CHRONIC HEV INFECTION?

Reduction in immune suppression alone can achieve cure/a sustained virologic response (SVR) in ∼30% of SOT recipients with chronic HEV infection, although this strategy can increase the risk of transplant rejection [18]. In patients with hematological malignancies, reducing immunosuppression is often not feasible and in certain instances has been associated with serious adverse outcomes including death [15]. A systematic review of ribavirin, the most extensively studied and widely used drug in the treatment of chronic HEV infection, found that cure of chronic HEV infection was achieved in 65% of individuals [19]. However, 28% required dose reductions due to ribavirin-induced anemia, which is of particular concern in patients with preexisting hematological disorders. In a European multicenter cohort study of HEV in patients with hematological malignancies, 45.5% of patients required ribavirin dose reductions [15]. Pegylated interferon-∝ (pegIFN-∝), alone or in combination with ribavirin, has been successfully used to treat a limited number of individuals with chronic HEV infection, but its risk of severe adverse effects including an increased risk of organ rejection among certain SOT recipients (particularly kidney and heart transplants) precludes its wider use [9].

The nucleotide NS5B RNA-dependent RNA polymerase inhibitor sofosbuvir, which is well established in the treatment of hepatitis C virus (HCV) infection, has demonstrated in vitro and in vivo activity against HEV replication, but this has not translated into clinical efficacy whereby SVR is achieved (when used at the HCV dose of 400 mg/d) either as monotherapy or in combination with ribavirin for chronic HEV infection, with the exception of 1 kidney transplant recipient cured with a combination of sofosbuvir and ribavirin after failing treatment with ribavirin monotherapy [20, 21]. Additionally, the emergence of resistance variants with reduced susceptibility to sofosbuvir has been observed in immunocompromised patients unsuccessfully treated for chronic HEV infection with sofosbuvir [22]. Higher doses of sofosbuvir (possibly for more prolonged durations) are yet to be explored in refractory chronic HEV infection.

There are several novel and preexisting compounds that have been found to have antiviral activity against HEV in vitro [23]. However, most are yet to transition into in vivo studies, and in some instances developmental progress has been hindered by concerns regarding toxicity. Silvestrol, a natural plant-derived compound, has shown particular promise and is one of the few compounds to date to progress to in vivo study demonstrating high potency against HEV genotype 1 with a reduction in viral titers in HEV-infected mice [23, 24]. Additionally, a pan-genotypic effect of silvestrol has been demonstrated in vitro against isolates from 4 different human HEV genotypes as well effective inhibition of an HEV strain harboring a mutation conferring ribavirin resistance [23, 24]. However, human studies of silvestrol are lacking. Although novel anti-HEV compounds show extremely promising preliminary experimental findings and there are no major toxicity concerns, wider enhanced efforts are needed to facilitate translation in clinical studies.

Initial current management of HEV infection in transplant recipients and other immunocompromised individuals should therefore be a reduction in immunosuppression, where possible [25]. Persistent HEV replication for >3 months (ie, chronic HEV infection) despite this warrants commencement of oral ribavirin for at least 12 weeks. In those with ribavirin-refractory HEV infection, PegIFN-∝ in combination with ribavirin (or possibly as monotherapy if ribavirin is not tolerated) could be considered. However, among SOT recipients, PegIFN-∝ use should only be considered in liver recipients, given the higher organ rejection risk in other SOT recipients. Long-term ribavirin at the lowest dose (to minimize adverse effects) able to suppress HEV replication could be considered for chronic HEV infection refractory to the above strategies, primarily aiming to reduce the risk of liver disease progression rather than aiming for HEV eradication/SVR [26].

## WHAT EVIDENCE EXISTS TO SUPPORT THE CLINICAL USE OF VIRUS-SPECIFIC T-CELL THERAPY, AND WHY MAY THESE THERAPIES BE OF PARTICULAR USE IN CHRONIC HEV INFECTION?

Recently, adoptive transfer of virus-specific T cells (VST) to restore antiviral immunity in severely immunocompromised individuals has shown great promise [27]. The most widely used strategy involves isolation and ex vivo or direct selection of VST from an HSCT donor or a third-party donor, followed by selective expansion of T cells directed against the viral target(s) of interest and subsequent infusion into the patient [27, 28]. VST derived from a healthy seropositive donor are typically selected to have a high degree of human leukocyte antigen matching, with the recipient thereby aiming to increase their persistence and antiviral effects and reduce the likelihood of unwanted effects such as graft-vs-host disease (GVHD). Over the last decade, this strategy in the context of treating viral infections has been used most frequently in the hematological setting following HSCT but also in SOT recipients to successfully treat a range of viral infections including cytomegalovirus (CMV), Epstein-Barr virus (EBV), BK polyomavirus (BKV), John Cunningham virus (JCV), and adenovirus infections, but it has not been used for HEV infection thus far [27, 29]. “Off-the-shelf” banks of third-party-derived cryopreserved VST available for a repertoire of viral infections are increasingly becoming established. Of note, however, is that the concentrations of VST can vary between productions. In a large study of compassionate access to VST therapy for adults and children, 65% (46/71) of patients with hematological malignancies who had active disease secondary to a viral infection (most commonly CMV) showed clinical improvement (based on viral load reduction and/or disease improvement) following receipt of VST [29]. Sixty-four percent of patients treated with VST therapy in their study were HSCT recipients, 22% were SOT recipients, and 14% had not undergone a transplant.

A potentially limiting factor for VST use in SOT recipients is the use of certain immunosuppressive agents such as the calcineurin inhibitor tacrolimus (which downregulates T-cell activation), which may hinder the expansion, persistence, and efficacy of third-party-derived VST infusions [29]. In vitro studies have already demonstrated that calcineurin inhibitors (tacrolimus and ciclosporin) and the mammal target of rapamycin (mTOR) inhibitor sirolimus potentiate HEV replication, whereas mycophenolic acid has an inhibitory effect [30, 31]. Mycophenolic acid and ribavirin have augmented anti-HEV effects when used in combination in vitro, but this effect was not observed when this strategy was adopted in vivo in transplant recipients [7]. Data on the precise effects these immunosuppressive agents have on HEV in the clinical setting are very limited and inconclusive thus far. Despite this, several reports exist demonstrating the clinical benefit of VST therapy in SOT recipients, although possibly in the context of dose reductions (or even withdrawal) of certain immunosuppressants [29, 32]. Overall, VST therapy has also been shown to be safe and well tolerated [27, 29, 32]. Infusion reactions can occur but are considered rare. There have been no reports of cytokine release syndrome or deaths associated with VST therapy [29]. There is a theoretical increased risk of GVHD in the post-transplant period due to alloreactive T cells attacking recipient tissues, and 1 study of prophylactic VST use after allogeneic HSCT reported increased GVHD rates (4 of 11 patients) [33]. However, this was not observed in the other reported studies of prophylactic VST use post-transplant [29, 33–37].

Another method for adoptive immunotherapy with VST is ex vivo engineering of autologous T cells to target a specific viral epitope [27, 28]. These engineered virus-specific T-cell receptor (TCR) T cells are typically monoclonal and monospecific. An advantage of this production method is that these virus-specific TCR T cells can more consistently be produced at high T-cell concentrations. Additionally, virus-specific TCR T cells can be modified to be resistant to immunosuppressive drugs [38, 39]. Hepatitis B virus (HBV)–specific TCR T cells have been successfully armored in liver transplant recipients to, albeit transiently, escape the immunosuppressive effects of tacrolimus and mycophenolate mofetil [38]. In a phase 1 dose escalation study of adoptively transferred HBV-specific TCR T cells in 8 patients with HBV-related hepatocellular carcinoma, TCR T-cell therapy was safe and well tolerated [40]. All 8 patients had either a reduction in circulating HBV DNA levels or their HBV DNA remained persistently undetectable in blood after receiving VST. Additionally, 7 of 8 patients experienced a decline or stabilization in serum hepatitis B surface antigen (HBsAg) levels. In another preliminary study by Soon et al., investigators collected and isolated CD8+ T cells from human subjects with both acute and chronic HEV infection and engineered the CD8+ T cells ex vivo to target specific HEV epitopes, and these redirected HEV-specific CD8+ TCR T cells subsequently demonstrated HEV-specific immunogenicity and cytotoxicity against target cells containing HEV epitopes [41].

A potential drawback of these epitope-specific TCR T cells is that there is a theoretical heightened risk for the potential emergence of escape variants [28]. Another consideration is that CD8+ T cells have been shown to be the predominant cell type in identified liver biopsies of patients with HEV-associated acute liver failure and therefore could also therefore play a role in the pathogenesis of severe liver injury [42]. Use of engineered HEV-specific TCR T cells poses a potential risk of uncontrolled proliferation and profound cytotoxicity [4].

Further investigation into the potential role of adoptively transferred HEV-specific T lymphocytes (both allogenic VST and HEV-specific TCR T cells) in chronic HEV infection is warranted. Determining the optimal concentrations of HEV-specific T cells infused, timing(s) of administration, potential combinations with other anti-HEV agents, and possible interactions with immunosuppressants will be future challenges if work in this area progresses.

## CONCLUSIONS

Novel immune-focused approaches for tackling chronic HEV infection are an extremely worthy avenue for exploration given the key role of T cells in achieving clearance of HEV, the immunodeficiency of the patients affected by chronic HEV, and the extremely limited antiviral repertoire to date. The clinical utility of VST therapy has been demonstrated in the treatment of a range other viral infections, and HEV-specific T-cell therapy could serve as a useful adjunct to the currently available strategies in the treatment of chronic HEV infection.

## Notes

***Financial support.*** No source of funding was required for this work.

***Potential conflicts of interest.*** No competing interests to declare.
